# Comparison of Performances of GeneXpert MTB/RIF, Bactec MGIT 960, and Bactec Myco/F Systems in Detecting Mycobacterium tuberculosis in Biopsy Tissues: a Retrospective Study

**DOI:** 10.1128/spectrum.01414-22

**Published:** 2023-05-08

**Authors:** Jiankang Zhao, Danni Pu, Yulin Zhang, Jiuxin Qu, Binghuai Lu, Bin Cao

**Affiliations:** a Laboratory of Clinical Microbiology and Infectious Diseases, Department of Pulmonary and Critical Care Medicine, Center of Respiratory Medicine, National Clinical Research Center for Respiratory Diseases, National Center for Respiratory Medicine, China-Japan Friendship Hospital, Beijing, China; b Institute of Respiratory Medicine, Chinese Academy of Medical Sciences, Beijing, China; c Graduate School of Peking Union Medical College, Chinese Academy of Medical Sciences, Peking Union Medical College, Beijing, China; d Department of Clinical Laboratory, Shenzhen Third People’s Hospital, Second Hospital Affiliated to Southern University of Science and Technology, Guangdong Provincial Clinical Research Center for Infectious Diseases (Tuberculosis), National Clinical Research Center for Infectious Diseases, Shenzhen, Guangdong, China; e Tsinghua University-Peking University Joint Center for Life Sciences, Beijing, China; f Department of Respiratory Medicine, Capital Medical University, Beijing, China; Houston Methodist Hospital

**Keywords:** *Mycobacterium tuberculosis*, biopsy tissue, GeneXpert, MGIT 960, Myco/F lytic system, Myco/F system

## Abstract

Tuberculosis remains a major global public concern as a leading cause of health care-associated infections. The detection of Mycobacterium tuberculosis (MTB) is challenging due to the paucibacillary nature of the pathogen. For suspected pulmonary and extrapulmonary tuberculosis patients, if sputum, bronchoalveolar lavage fluid (BALF), related samples are negative for MTB, or suspected tumors, biopsy tissues may provide a better diagnostic yield. This study was aimed at comparing the performances of three methods in identifying MTB in biopsy tissues, including the Bactec mycobacterial growth indicator tube 960 (MGIT 960) system, the GeneXpert MTB/RIF assay (GeneXpert), and the Bactec Myco/F lytic culture (Myco/F) system. Biopsy samples from 3,209 nonduplicated patients were retrospectively enrolled between January 2018 and September 2021, of which 180 (5.6%) were positive for MTB by at least one method. GeneXpert revealed the highest recovery rate (134/162, 82.7%), followed by MGIT 960 (99/135, 73.3%) and Myco/F (26/143, 18.1%), and the composite positive rate for GeneXpert and MGIT 960 was 96.6% (173/179). Pairwise comparisons were conducted after completion of both tests, and the results showed that Myco/F had significantly lower detection rates than GeneXpert and MGIT 960 (16.4% versus 82.8%, *P < *0.001; 14.3% versus 71.4%, *P < *0.001). In summary, GeneXpert was the most sensitive and recommended method for MTB detection in biopsy tissues, and the combination of GeneXpert and MGIT 960 could improve the overall diagnostic yield.

**IMPORTANCE** Mycobacterium tuberculosis (MTB) poses a severe threat to public health worldwide. The diagnosis of tuberculosis is challenging due to the low load of the microorganism in samples. Biopsy tissues are sometimes collected via invasive procedures with limited size, and additional samples are often inaccessible. The GeneXpert MTB/RIF assay, Bactec MGIT 960 system, and Bactec Myco/F lytic system have been used in detecting MTB in our laboratory. Here, we evaluated the performances of these three methods in 3,209 biopsy tissues samples to establish a more effective protocol based on clinical requirements. Attempts for a locally optimized protocol should be always made.

## INTRODUCTION

Tuberculosis remains a challenging public health problem, especially in low- and middle-income countries (1, 2). According to a recent World Health Organization report, the incidence of this epidemic disease in China was 59 per 100,000 in 2020, accounting for 8.5% of the global total (2). In addition, China is on the list of high-burden countries for tuberculosis from 2021 to 2025 (2).

The disease spreads mainly through inhalation and manifests as pulmonary tuberculosis. The early and accurate etiological diagnosis of tuberculosis is crucial for its prevention and treatment (3). However, this is sometimes challenging due to the paucibacillary load of Mycobacterium tuberculosis (MTB) in clinical samples (4). Biopsy tissue is commonly used for diagnosing lung cancer in clinical practice (5). To date, several studies have used biopsy tissue for the diagnosis of pulmonary and pleural tuberculosis and have obtained positive results (6–10). Biopsy tissue is a promising specimen for subjects without positive etiological findings from routine respiratory samples (e.g., sputum, bronchoalveolar lavage fluid [BALF]), especially when a malignant disease cannot be excluded. However, due to the side effects of invasive examination, such as local pain and percutaneous emphysema (10), a laboratory protocol with good performance should be established for MTB identification.

Several tests are currently used for processing biopsy tissue samples to establish a diagnosis of tuberculosis. The GeneXpert MTB/RIF system (GeneXpert; Cepheid, Inc., Sunnyvale, CA, USA), a rapid and automated system based on real-time PCR and molecular beacon technology, can detect MTB and rifampin resistance simultaneously in sputum, BALF, biopsy tissues, pleural effusion, paraffin-embedded tissues, and extrapulmonary tuberculosis specimens with high sensitivity and specificity in 2 h (1, 11–13). The Bactec MGIT 960 system (MGIT 960) is an automated system for the growth and detection of MTB and nontuberculosis mycobacteria (NTM) in a 7-mL culture tube. A mixture of antibiotics that included polymyxin B, amphotericin B, nalidixic acid, trimethoprim, and azlocillin (PANTA) was added to the tube to increase the positive rate of this system (14, 15). In addition, the Bactec Myco/F lytic culture system (Myco/F) is also an automated, continuous-monitoring system for fungal and mycobacterial culture of blood and sterile body fluid specimens (16–18). The positive yield of culture establishes definitive proof of tuberculosis, but it is time-consuming and the time to positivity averages 14 to 28 days.

Our laboratory has extensive experience in the use of the above three methods for detecting etiological agents. Here, we initiated a retrospective single-center study to better assess the performance of these methods for detecting MTB in biopsy tissue specimens based on the protocol developed in our laboratory.

## RESULTS

### Performance of the three methods in the detection of MTB.

**FIG 1:**
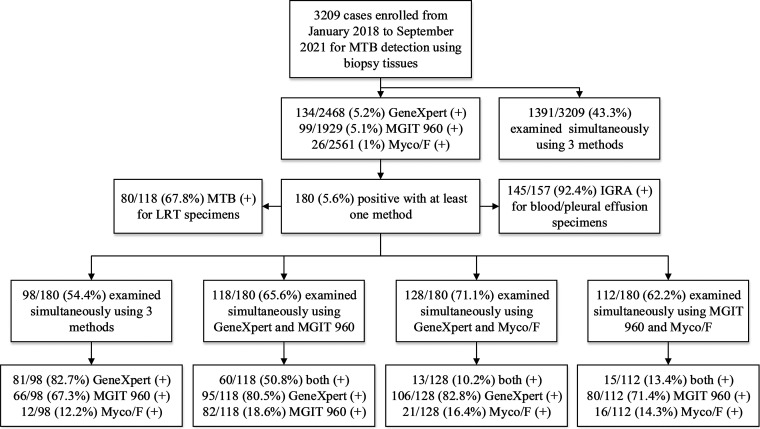
Diagnostic accuracy of GeneXpert, Myco/F, and MGIT 960 for detection of MTB from suspected tuberculosis patients based on retrospective data.

A total of 3,209 nonduplicated biopsy tissue specimens, each approximately 0.5 to 2 mm^2^, were collected from the same number of patients (Fig. 1). Apart from 180 bacteriologically confirmed tuberculosis cases, there were pleuropulmonary and bronchial infections (780 cases, 23.3%), lung cancer (546, 17.0%), idiopathic interstitial lung disease (305, 9.5%), lymphoma and other hematological diseases (28, 0.9%), and others. The characteristics of the enrolled patients are summarized in Table 1. Of the 3,209 specimens, 2,468, 1,929, and 2,561 were sent for GeneXpert, MGIT 960, and Myco/F assays, and the MTB-positive rates were 5.2% (134/2,468), 5.1% (99/1,929), and 1.0% (26/2,561), respectively. Furthermore, 12 NTM strains were detected by MGIT 960 (0.6%, 12/1,929), including M. avium complex (*n* = 9), M. fortuitum (*n* = 1), M. abscessus (*n* = 1), and M. kansasii (*n* = 1). The ratio of MTB/NTM was approximately 8.3:1 (99/12).

**TABLE 1:** Characteristics of suspected and confirmed tuberculosis patients based on biopsy tissue samples

Characteristic^*a*^	Value^*b*^ for:	
	All patients (*n* = 3209)	Patients with tuberculosis (*n* = 180)
Age, median (range) (yr)	60 (10–105)	49 (17–86)
Gender		
Male	1,898 (59.1)	101 (56.1)
Female	1,311 (40.9)	79 (43.9)
Biopsy site		
Lung	2,805 (87.4)	165 (91.7)
Pleura	191 (6)	6 (3.3)
Lymph node	73 (2.3)	5 (2.8)
Bronchi	43 (1.3)	3 (1.7)
Others	97 (3)	1 (0.6)
MTB^+^ for LRT specimens	142/1,845 (7.7)	70/104 (67.3)
IGRA^+^ for blood/pleural effusion	1,143/2,548 (44.9)	145/157 (92.4)

a MTB, Mycobacterium tuberculosis; LRT, lower respiratory tract; IGRA, interferon gamma release assay.

b Values are number (%) of patients unless otherwise indicated.

According to the retrospective survey results, a total of 1,391 biopsy tissue samples underwent all three tests simultaneously; of these, samples from 98 patients were positive for at least one of the three methods. Compared with the culture results of MGIT 960, the sensitivity, specificity, positive predictive value, and negative predictive value of GeneXpert were 75.8% (95% confidence interval [CI], 63.4 to 85.1%), 97.7% (95% CI, 96.7 to 98.4%), 61.7% (95% CI, 50.2 to 72.1%), and 98.8% (95% CI, 98 to 99.3%), respectively. Meanwhile, the above-mentioned values of Myco/F were 16.7% (95% CI, 9 to 28.3%), 100% (95% CI, 99.5 to 100%), 91.7% (95% CI, 59.8 to 99.6%), and 96% (95% CI, 94.8 to 97%), respectively.

In addition, biopsy tissue specimens from 180 of 3,209 (5.6%) patients were positive for at least one of the three tests and were defined as bacteriologically confirmed tuberculosis cases. Furthermore, of the above 180 cases, 162 were tested by the GeneXpert assay, with a positive rate of 82.7% (134/162). Similarly, the positive rates of MGIT 960 and Myco/F were 73.3% (99/135) and 18.2% (26/143), respectively. Of the 117 specimens that were negative by Myco/F, 27 were positive for fast-growing bacteria or yeasts, including *Moraxella* (*n* = 1), *Candida* (*n* = 2), Staphylococcus (*n* = 5), *Enterobacteria* (*n* = 3), Streptococcus (*n* = 10), *Neisseria* (*n* = 1), Acinetobacter (*n* = 1), *Kocuria* (*n* = 1), and Pseudomonas (*n* = 3), of which 2 S. aureus isolates and 3 enterobacteria were considered to be dual infections according to the patients’ clinical conditions.

Of the 180 cases, samples from 98 were sent for all three tests, and the positive rates for GeneXpert, MGIT 960, and Myco/F were 82.7% (81/98), 67.4% (66/98), and 12.2% (12/98), respectively. Notably, 31 (31.6%), 14 (14.3%), and 1 (1%) patient samples were positive only by GeneXpert, MGIT 960, and Myco/F, respectively.

Pairwise comparative analysis was conducted after completion of both tests. Of the 180 MTB-positive patients, 118 were simultaneously tested for MTB by GeneXpert and MGIT 960, and 95 (80.5%) and 82 (69.5%) were positive by each method, respectively, while 60 (50.9%) were positive for both. The performances of the two methods were comparable (*P = *0.071). Further, 128 and 112 samples were simultaneously sent for GeneXpert and Myco/F assays and for MGIT 960 and Myco/F culture, respectively. Myco/F yielded significantly lower recovery rates than GeneXpert and MGIT 960 (16.4% versus 82.8%, *P < *0.001; 14.3% versus 71.4%, *P < *0.001). Furthermore, 179 of the 180 samples were sent for either GeneXpert or MGIT 960 detection, with 173 positive tests (96.6%).

### Detection results of lower-respiratory-tract (LRT) specimens.

Of the 180 patients with MTB-positive biopsy specimens, 118 patients had sent their sputum or BALF specimens for GeneXpert or MGIT 960 assay within 1 month before and 1 month after the biopsy examination, and of these, 67.8% (80/118) were MTB positive. Meanwhile, 8 of the 80 patients were sent for tissue biopsy detection 1 to 13 days after their respiratory tract samples were MTB positive.

### IGRA results of whole-blood or pleural effusion samples.

Of the 180 MTB-positive patients, 157 underwent interferon gamma release assays (IGRAs) within 1 month before and 1 month after the biopsy examination, including 8 pleural effusion samples and 149 whole-blood samples, of which 92.4% (145/157) were IGRA positive.

## DISCUSSION

Before the global outbreak of COVID-19, tuberculosis was the leading cause of death from a single infectious disease worldwide, especially in developing countries (1). Rapid and accurate detection of MTB is essential for allowing appropriate initiation of the treatment, yet 40% of estimated tuberculosis cases were missed from diagnosis and reporting (1). Biopsy tissues obtained via invasive procedures have been used for tuberculosis diagnosis. Previous studies comparing the performances of GeneXpert and MGIT 960 in diagnosing tuberculous pleuritis and extrapulmonary tuberculosis in biopsy samples did not show a substantial discrepancy, which could be explained by the small sample sizes and differences in preprocessing methodologies (6, 9, 12). In this study, we assessed the performance of GeneXpert, MGIT 960, and Myco/F in detecting MTB in biopsy specimens.

**FIG 2:**
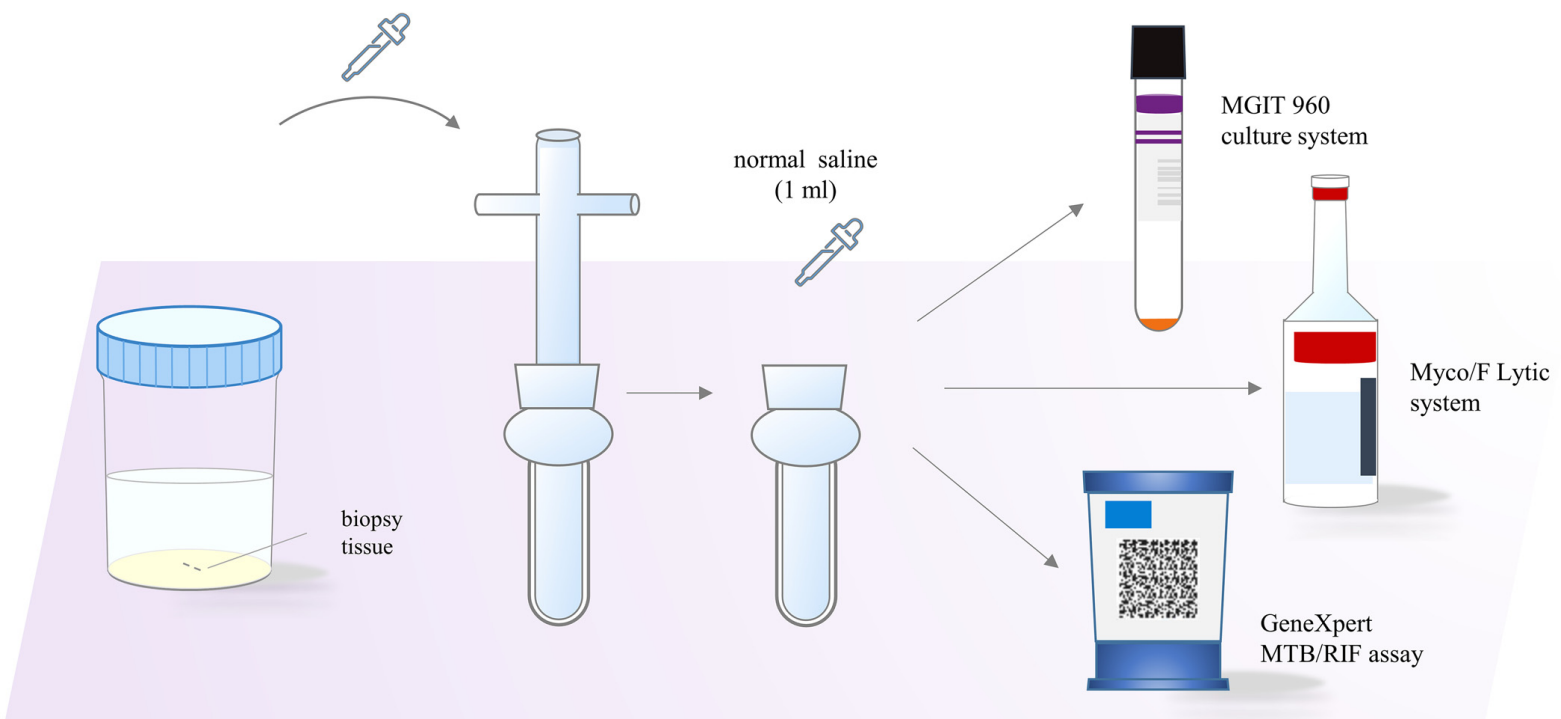
Schematic diagram of MTB identification in biopsy tissue samples by the molecular method (GeneXpert) and by mycobacterial culture (Myco/F and MGIT 960).

The GeneXpert system, capable of rapidly identifying MTB with rifampin (RIF) resistance within 2 h, has been widely investigated using a wide variety of samples, including paraffin-embedded tissues (11), blood samples (19), sterile body fluids (20), bones (21), and extrapulmonary samples (13, 22), thus proving that GeneXpert is a valuable tuberculosis diagnostic tool. In comparison, the culture of MTB requires a longer cycle (usually 14 to 28 days). Our results suggested that the GeneXpert system was a superior diagnostic tool for MTB detection in biopsy tissue samples compared to MGIT 960 and Myco/F, based on the protocol described in Fig. 2. Although a mixture of antibiotics was added to the MGIT 960 bottle, we found that there were still fast-growing, antimicrobial-resistant bacteria in some cases that can interfere with the detection of MTB, which may partially explain why the positive rate of MGTI 960 was lower than that of GeneXpert.

In addition, the combined use of GeneXpert and MGIT 960 yielded a sensitivity of 96.6% (173/179), proving that this combination could improve the overall diagnostic yield. Besides, MGIT 960 detected 12 NTM cases (12/1,929, 0.6%) and could also compensate for the disadvantage that GeneXpert cannot distinguish between dead and live mycobacteria.

The Myco/F lytic system was also used for MTB detection in sterile specimens (18, 23). Wang et al. documented that of 103 culture-positive sterile body fluid samples, the recovery rate was 86.4% (89/103) for the Myco/F lytic system and 75.7% (78/103) for MGIT 960 (18). Harausz et al. documented that the specificities and sensitivities of MGIT 960 for the recovery of MTB from pleural fluid are not statistically different from those of Myco/F (17). According to the above-mentioned studies, the Myco/F lytic system was a promising alternative to MGIT 960 for diagnosing MTB in sterile body fluids. However, in the present study, the Myco/F system showed a significantly lower recovery rate (*P < *0.001) than that of MGIT 960. The biopsy tissues were ground, homogenized, and equally distributed into the two systems for MTB culture. The discrepancies might be explained by the fast-growing bacteria or fungi that mask the growth of MTB in the Myco/F culture system and the differences in additives between the two methods. The Myco/F lytic culture is supplemented with Middlebrook 7H9 and brain heart infusion broth, a nonselective culture medium intended to be an adjunct to the aerobic blood culture medium to improve the recovery of mycobacteria, yeasts, and fungi from clinical samples. The MGIT 960 culture contains modified Middlebrook 7H9 broth supplemented with a mixture of antibiotics (PANTA) and oleic acid-albumin-dextrose-catalase (OADC) (a mycobacterial growth supplement), which increases the recovery rate (14, 17). Considering all this together, the Myco/F culture was less effective in recovering MTB from biopsy specimens.

Sputum/BALF specimen collection is less invasive than biopsy specimen collection, but the MTB recovery rate for sputum/BALF specimens was lower than that for biopsy specimens (67.3% versus 96.6%). The IGRA has also been extensively applied as a diagnostic tool for pulmonary tuberculosis, articular tuberculosis, tuberculous peritonitis, tuberculous pleurisy, and tuberculous meningitis in both peripheral blood and extrapulmonary samples (24–27). A previous study investigated the value of IGRA in the diagnosis of bacterium-negative pulmonary tuberculosis and found that the sensitivity rate of IGRA was 84.9% (28). Our study obtained a similar result. Among the 180 MTB-positive patients, 157 were sent for IGRA and 92.4% (145/157) were positive, revealing that IGRA can serve as a sensitive supplementary tool for tuberculosis screening.

The present study is limited by several factors. First, this is a single-center, retrospective study. Some parameters were inaccessible, and not all patients underwent the above three tests simultaneously. Therefore, the conclusions might be biased, which is not uncommon for this type of study design, and should be interpreted with caution and further validated elsewhere. Second, this laboratory protocol has a relatively high requirement for aseptic conditions, and the degree of sample grinding may affect the positive rate of tests due to the heterogeneous distribution of MTB in histological sections (29). Third, tissue sampling is an invasive procedure and the sample volume is limited, and thus the patient’s clinical conditions, financial burden, and laboratory testing capacity should be comprehensively considered before all three tests are recommended. Finally, the GeneXpert MTB/RIF Ultra has been developed, based on GeneXpert MTB/RIF, to increase the positivity rate of MTB by using two additional multicopy targets (*IS6110*, *IS1081*) and four *rpoB*-specific probes (29, 30). This was unavailable in low- and middle-income countries, including China. More efficient tissue biopsy specimen processing methods should be explored and evaluated for a better recovery rate. Attempts for locally optimized protocols should always be made.

In summary, we compared the performances of three methods for MTB recovery from biopsy tissue samples, and the data demonstrated that GeneXpert had superior sensitivity and that the combined application of GeneXpert and MGIT 960 could improve the overall yield. This study will help improve the diagnosis of bacteriologically confirmed tuberculosis and aid decision-making in the context of initial treatment.

## MATERIALS AND METHODS

### Ethics statement.

Permission for using the information in the medical records of the patients for research purposes was granted by the Ethics Committee of the China-Japan Friendship Hospital (CJFH) (2022-KY-133).

### Study design and population.

The data in this study were retrospectively recovered from the medical record system and laboratory information system of CJFH, National Center for Respiratory Medicine, a 2,100-bed tertiary teaching medical center in Beijing, China, between 1 January 2018 and 12 September 2021. Inclusion criteria were as follows. (i) Patients were included if suspected of having pulmonary tuberculosis or extrapulmonary tuberculosis and had the following conditions: symptoms or signs consistent with tuberculosis (cough > 2 weeks, fever, night sweats, unintentional weight loss, hemoptysis, shortness of breath, chest pain, lymphadenopathy, or pleural effusion), an abnormal chest radiograph compatible with tuberculosis (pulmonary infiltrates, intrapulmonary mass, cavitary, etc.), were treated empirically with antituberculosis chemotherapy, or had no improvement on antibiotics. (ii) Biopsy samples were sent for at least one of the three MTB detection methods for the diagnosis and differential diagnosis of tuberculosis. (iii) The basic medical and microbiological data, including the age, gender, clinical diagnosis, and biopsy sites of the patients, and the methods and results of MTB detection were accessible from the electronic medical record system and laboratory information system of the CJFH hospital.

The biopsy tissues were mainly collected from the lung and less frequently from the pleura, peritoneum, lymph nodes, and bronchi. The MTB test results for sputum and BALF specimens, as well as interferon gamma release assay (IGRA) results for whole-blood/pleural effusion samples, are detailed in Table 1. A total of 3,209 patients who were sent for MTB detection using biopsy tissues were enrolled in this study; they ranged in age from 10 to 105 years, with a mean age of 60 years, and 40.9% (1,311/3,209) were female.

### Protocol for biopsy tissue processing: microbiological culture and GeneXpert assay.

The biopsy tissues from different biopsy sites were collected in normal saline after puncture surgery, stored at room temperature, and processed within 2 h in accordance with the protocol shown in Fig. 2. Briefly, tissue biopsy specimens were ground and homogenized in a 10-mL glass container and grinder (Naitong Industrial Products Co., Ltd., Guangzhou, China) due to the heterogeneous distribution of MTB in histological sections. If necessary, long or large tissues were minced with sterile scissors or scalpel blades. Afterwards, 1 mL sterile saline was added and mixed thoroughly. Then, equal amounts of the suspension were transferred into sterile vials and stirred gently. Finally, the suspension in each vial was thoroughly transferred into (i) a Myco/F lytic culture bottle, (ii) an MGIT 960 tube and decontaminated before liquid culture with *N*-acetyl-l-cysteine-sodium hydroxide (NALC-NaOH) provided by the manufacturer, and/or (iii) a GeneXpert reaction cartridge for rapid molecular MTB detection, respectively. The recommended sample sizes for Myco/F, MGIT 960, and GeneXpert are 1 to 5, 0.5, and 2 mL, respectively. Therefore, in the present study, we supplemented samples to the recommended volume with sterile normal saline for the GeneXpert assay. The positive cultures by Myco/F or MGIT 960 were verified by Zeihl-Neelsen (ZN) staining, to examine the presence of acid-fasting bacilli (AFB) under oil immersion (100×) using a light microscope, and by a TaqMan real-time PCR. Specific primers and probes of the latter were designed for MTB and mycobacteria using genomic DNA as a template according to the instructions of the tuberculosis and nontuberculous mycobacterial real-time PCR detection kit from CapitalBio Technology, Inc. (Beijing, China). The criterion for determining smear positivity was to observe positive AFB by ZN microscopic examination. If no detectable growth was observed after 42 days of incubation, the culture was defined as negative.

### Case definitions.

In this retrospective study, MTB positivity was defined as positive results for at least one of the three tests, namely, GeneXpert, MGIT 960, or Myco/F. Patients with both positive MTB results and abnormal radiological chest imaging were defined as bacteriologically confirmed tuberculosis cases.

### Statistical analysis.

Statistical analysis was done by using SPSS Statistics version 21 (IBM). The comparison of positive rates was done using the χ^2^ test. A *P* value of <0.05 was considered statistically significant.

### Data availability.

The original data presented in the current study are all included in the article. Further inquiries can be directed to the corresponding author.
